# An Overview of Tuberculosis-Designated Hospitals in China, 2009-2015: A Longitudinal Analysis of National Survey Data

**DOI:** 10.1155/2019/9310917

**Published:** 2019-08-20

**Authors:** Yuhong Liu, Yu Pang, Jian Du, Wei Shu, Yan Ma, Jingtao Gao, Lijie Zhang, Shaofa Xu, Liang Li

**Affiliations:** ^1^Clinical Center on TB Control, Beijing Chest Hospital, Capital Medical University/Beijing Tuberculosis & Thoracic Tumor Research Institute, Beijing 101149, China; ^2^National Clinical Laboratory on Tuberculosis, Beijing Chest Hospital, Capital Medical University/Beijing Tuberculosis & Thoracic Tumor Research Institute, Beijing 101149, China

## Abstract

**Design:**

A national tuberculosis- (TB-) designated hospital survey was conducted in 2015 to identify significant changes since 2009 in implementation of TB-testing services within hospitals of various types and administrative levels in various regions in China.

**Methods:**

In 2015, all TB-designated hospitals were required to complete questionnaires designed by the National Clinical Center for TB. Community hospitals also completed simplified questionnaires as part of the study.

**Results:**

Overall, in 2015 there were 1685 TB-designated hospitals in China, consisting of 1335 (79.2%) county-level hospitals and 350 (20.8%) hospitals at the prefecture level and above. The percentage of counties with TB-designated hospitals in the western region (57.4%) was significantly lower than corresponding percentages for eastern and middle regions (70.3% and 96.5, respectively). Based on data recorded on hospital surveys in both 2009 and 2015, significant differences were noted between years in proportions of general hospitals with TB wards and of specialized infectious disease hospitals (*P *< 0.01). Of 1256 county-level laboratories conducting smear microscopy, only 979 (79%) performed external quality control evaluations of test results in 2015. For prefecture-level hospitals, 70% (234/334), 76% (155/203), and 67% (66/98) of hospitals obtained external quality control validations of smear microscopy, phenotypic DST, and molecular test results, respectively.

**Conclusions:**

Although China's health reform efforts have resulted in improved TB patient access to quality health care, more attention should be paid to balancing the distribution of medical facilities across different regions. In addition, laboratory capabilities and quality control systems should be strengthened to ensure delivery of high-quality laboratory services by TB-designated hospitals throughout China.

## 1. Introduction

Tuberculosis (TB) remains a leading cause of morbidity and mortality worldwide [1, 2]. In 2017, an estimated 10.0 million incident cases of tuberculosis were detected that resulted in deaths of 1.6 million people from the disease [1]. In many counties, hospitals are usually the major providers of health care to TB patients [3–5]. Unfortunately, most hospitals in Asian and African countries often function outside of National TB Control Program (NTP) supervision, resulting in hospital delivery of TB services that are often unsatisfactory [3, 6]. To address this concern, the World Health Organization (WHO) has called for participation of all health care providers in the development of improved practices to deliver comprehensive services for TB prevention and control [7].

China currently has the second highest TB burden in the world, accounting for 11% of all cases globally [1]. However, China has achieved impressive reductions in TB prevalence and mortality over the last two decades, as demonstrated by results of the Fifth National TB Epidemiological Survey conducted in 2010. This decrease in TB burden has been mainly attributed to significant improvement in treatment outcomes driven by the major shift within the past decade from hospitals to public health centers as providers of “directly observed treatment, short-course” (DOTS) TB treatment [2]. Despite this success, the important role of hospitals in delivering TB testing and treatment services in China cannot be ignored [8, 9]. In fact, a recent national TB survey in China demonstrated that approximate nine-tenths of TB patients visited hospitals first, of which only one quarter of hospitalized suspected TB cases were referred to TB dispensaries [3]. As a consequence, the NTP has been moving TB care towards a hospital-based health care model, which since the 2010s has mandated that only hospitals specializing in TB diagnosis and treatment provide care to TB patients [8]. This model differs from the public-private mixed model used in other regions that is based on participation of all relevant public and private health care providers for implementing a unified TB treatment and control system. In the hospital-based health care model, only public hospitals designated by a local authority are integrated within the national TB control program that clinically manages all TB patients throughout China [3]. Moreover, in this model TB patients are diagnosed and treated in TB-designated hospitals, while the public health sector provides patient follow-up health care services. Indeed, this model has already been used with success, as TB-designated hospitals have played an essential role in the already apparent effectiveness of TB control strategies implemented in China since the reform of the TB control model. However, in spite of this early success, knowledge related to the impact of reforms remains limited. Therefore, to fill this information gap, two national TB-designated hospital surveys were administered across 31 Chinese administrative regions in 2009 and 2015 to collect data for later comparison of results between years. In this way, an assessment could be made of the progress made towards integrating TB services into health care delivery by TB-designated hospitals. Ultimately, the comparison of results for 2015 to those already reported for 2009 [9] should identify challenges faced by the TB-designated hospital system in implementing TB control programs in China.

## 2. Methods

### 2.1. Setting and Data Collection

The national TB-designated hospital survey was conducted across 31 Chinese administrative regions in 2015. All TB-designated treatment hospitals were required to complete the questionnaire designed by the National Clinical Center for TB in China. Specific information within the questionnaire requested from each prefecture-level hospital included (i) a basic overview of the hospital; (ii) human resources; (iii) income and expenses; (iv) diagnosis and management of TB patients; (v) TB laboratory settings; (vi) infection control measures. Meanwhile, simplified questionnaires were used for the collection of basic information from county hospitals to provide a basic overview of each hospital. These questionnaires were distributed to the provincial health administration, which supervised their distribution to and collection from TB-designated hospitals in local administrative regions. All staff who engaged in the completion of questionnaires attended a training course held by the National Clinical Center for Tuberculosis before the start of the study. In order to confirm data quality, 20% of the questionnaires were rechecked against original hospital records by staff from each relevant local provincial bureau of health. Any discrepant data were addressed via telephone interview.

## 3. Definitions

All hospitals in China are classified into three administrative assignment levels (tertiary, secondary, or primary) as determined by the local health bureau. Tertiary care hospitals have more than 500 beds; secondary care hospitals have 100-499 beds, and primary care hospitals have fewer than 100 beds [9].

The hospitals were also classified according to function as follows: a TB-designated (specialized) hospital is defined as a hospital that provides health care only for tuberculosis cases; an infectious diseases hospital provides care for TB cases and other infectious disease cases, such as cases involving hepatitis, HIV, etc.; a general hospital is defined as hospital that provides health care for treating various diseases, but with a dedicated ward for care of TB patients.

According to established Chinese administrative divisions, eastern region provinces include Liaoning, Hebei, Beijing, Tianjin, Shandong, Jiangsu, Zhejiang, Shanghai, Fujian, Guangdong, and Hainan; middle region provinces include Heilongjiang, Jilin, Shanxi, Henan, Hubei, Jiangxi, Anhui, and Hunan; western region provinces include Shaanxi, Inner Mongolia, Guangxi, Gansu, Qinghai, Ningxia, Xinjiang, Sichuan, Chongqing, Yunnan, Guizhou, and Xizang.

## 4. Data Analysis

Questionnaire data were entered in duplicate by two different survey staffs using EpiData version 3.1. After data entry, data were transferred to SPSS version 19.0 (SPSS Inc., Chicago, IL, USA) for data management, cleaning, and analysis. The chi-square test was performed to compare proportions of hospitals with different geographic and administrative level characteristics with regard to general characteristics, hospital specialty, laboratory testing capabilities, quality control, patient follow-up, and other characteristics. A difference was declared significant for* P* < 0.05.

## 5. Results

### 5.1. Geographic Distribution of TB-Designated Hospitals

During the study, a total of 1685 TB-designated hospitals were operating in China, consisting of 1335 (79.2%) hospitals at the county level and 350 (20.8%) hospitals at the prefecture level and above. Of 2869 counties, 46.5% hosted local TB-designated hospitals, with the distribution of TB hospitals exhibiting significant differences among distinct regions (*P *< 0.01). As summarized in Table 1, the percentage of counties with TB-designated hospitals in the western region (40.0%) was significantly lower than corresponding percentages in eastern (52.1%) and middle (49.0%) regions (*P *< 0.01). At the prefecture level and above, 350 of 492 (71.1%) prefectures hosted local TB-designated hospitals. The middle region had the highest percentage of prefectures with TB-designated hospitals (96.5%), a percentage that was significantly higher than corresponding percentages in eastern (70.3%) and western (57.4%) regions (*P *< 0.01).

Surveyed hospitals had a total of 32,103 beds for treatment of TB cases (Table 2). We further analyzed the distribution of TB beds according to population size and estimated TB case numbers for different regions. The number of TB beds per 100,000 population was 2.41 overall and ranged from 2.13 in the western region to 2.78 in the middle region. Calculation of number of TB beds per 1000 patients, as used in the Fifth National TB Prevalence Survey in 2010, yielded a value of 5.25 TB beds per 1000 patients across China, with highest and lowest numbers corresponding to 7.91 and 3.07 TB beds per 1000 patients for eastern and western regions, respectively.

**Figure 1:**
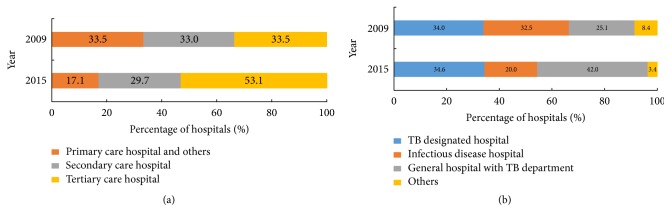
*Classification of prefectural TB-designated hospitals*. (a) The prefectural TB-designated hospitals stratified to hospital level; (b) the prefectural TB-designated hospitals stratified to hospital type.

Of 350 prefecture-level TB hospitals, 186, 104, and 60 were classified as tertiary care, secondary care, and primary care and other hospitals (Figure 1). Statistical analysis revealed that there was a significant increase in the percentage of tertiary care hospitals among prefecture-level TB hospitals (*P *< 0.01) between 2009 and 2015, of which major hospital types observed were specialized TB hospitals and general hospitals with TB wards, accounting for 34.6% and 42.0% of total prefecture-level TB-designated hospitals, respectively. When comparing observed distributions of various hospitals by type between 2009 and 2015, a significant difference between years was noted in proportions of general hospitals with TB wards and in proportions of infectious disease hospitals (*P *< 0.01).

### 5.2. Overview of Prefecture-Level Hospital TB Laboratories

Of 350 prefecture-level TB-designated hospitals surveyed, 334 (95%) had the capacity to perform smear microscopy in 2015. In fact, 3,622,104 smear tests obtained from presumptive TB patients were performed by prefecture-level TB hospitals as the most common diagnostic TB test provided by these hospitals (Table 3). Meanwhile, 241 (69%) of 350 TB-designated hospitals had the capacity to carry out mycobacterial culture testing in 2015 (regardless of culture method used), a rate 1.9-fold higher than the rate obtained in 2009. The number of mycobacterial culture tests in 2015 was 898,176, an increase of 126% over the number of tests in 2009. Notably, the number of TB-designated hospitals capable of performing phenotypic DST services in 2015 was only 1.6 times the corresponding number in 1999; however, from 2009 to 2015, the annual number of DST services performed increased by 971% (53,518 in 2009 versus 626,832 in 2015) and the average number of tests per hospital in 2015 was 6.2 times greater than in 2009. With regard to molecular testing, only 85 hospitals (24% of the total) were able to conduct GeneXpert in 2015, with a test volume of only 33,264 tests processed that year.

### 5.3. Quality Control of Laboratory Tests

An overview of TB laboratories incorporating quality control measures for evaluation of laboratory test results is presented in Table 4. Of 1256 county-level laboratories conducting smear microscopy, only 979 (79%) incorporated external quality control evaluation of test results in 2015 overall, while corresponding percentages ranged from 71% (238/336) in the western region to 87% (408/475) in the eastern region. Regarding prefecture-level hospital smear microscopy results, 70% (234/334), 76% (155/203), and 67% (66/98) of hospitals incorporated external quality control measures for evaluation of smear microscopy, phenotypical DST, and molecular methods results, respectively. In addition, a significantly higher proportion of prefecture-level hospitals in the eastern region than in the western region incorporated external quality control measures to evaluate smear microscopy results (*P *= 0.001).

## 6. Management of TB Patients

After completion of treatment in TB-designated hospitals, patients must continue their TB treatment for multiple months during the follow-up period, prompting us to analyze TB patient management strategies used by prefecture-level TB-designated hospitals. As shown in Table 5, a mixed pattern of hospital-based and Centers for Disease Control- (CDC-) based follow-up was predominantly adopted by prefectural-level hospitals in China, of which 54% used this mixed follow-up strategy. More specifically, approximately one quarter of TB hospitals referred patients to the local CDC for follow-up, while the other 76 (21.71%) of hospitals conducted follow-up themselves. Notably, TB patients from 4 (1.1%) prefecture-level TB hospitals were lost to follow-up.

## 7. Discussion

The Chinese government has been working to tackle tuberculosis during the past half-century [10]. Since the 2010s, a revised model has been widely implemented for building a hospital-based health care system to achieve TB control in China [8]. In this study, we systematically describe the dynamic pace of change in TB hospital-based TB control efforts by comparing 2015 results with those obtained in 2009. Our results demonstrate that approximately half of counties and 70% of prefectures hosted TB-designated hospitals in 2015. However, in spite of the sustained increase in the number of TB-designated hospitals, central issues still exist, including a shortage of hospital beds and unbalanced distribution of medical facilities across regions. With respect to the shortage of beds, this major challenge reflects a lack of access to health services that is especially pronounced within numerous counties and prefectures that lack TB-designated hospitals. Lack of access has the greatest impact on the poorest and most vulnerable patients, who must often travel to other regions to obtain treatment, a practice that promotes ongoing TB transmission within the community [11]. With respect to unequal distribution of medical facilities, in a recent national TB prevalence survey, the western region was considered to be a TB hotspot, with the highest TB prevalence rates in China [3]. Notably, significantly lower percentages of counties and prefectures with TB hospitals were found in the western region than in eastern and middle regions. Similarly, the number of TB beds per 1,000 estimated TB cases was lower in the western region than in the eastern and middle regions. This unbalanced distribution of TB facilities reflects the inequality of regional economic development in China, as well as the fact that funding for improvement of TB facilities mainly relies on local budgets [12]. Thus, these findings highlight the urgent need for greater allocation of national funds to resource-limited settings that lack funding for TB program delivery.

The TB laboratory plays a major role in early detection of TB cases and timely initiation of treatment, thus preventing TB transmission within the community [13]. Our study demonstrated that between 2009 and 2015 there was a significant increase in overall demand for smear microscopy, culture testing, and phenotypic drug susceptibility testing services performed by prefecture-level laboratories, thus highlighting the increasing importance of TB-designated hospitals in meeting this growing need. Of note, the greatest increase was observed in the volume of phenotypic drug susceptibility tests performed, indicating rapid scale-up of capacity for detection of drug-resistant TB cases in prefecture-level hospitals. Despite these increases, we also acknowledge several key challenges ahead. First, smear microscopy remains the cornerstone of TB diagnosis, especially in resource-limited settings [14]; however, 5% of prefecture-level laboratories could not perform smear microscopy, with similar results observed for culture testing and phenotypic DST. These results are of concern, since prefecture-level TB hospitals have been designated to provide health care services to drug-resistant TB patients. Thus, our findings suggest that the government should declare that hospitals must possess minimum laboratory capabilities as part of mandatory fundamental requirements they must meet before functioning as TB-designated hospitals. Second, in recent years the WHO has endorsed use of commercial GeneXpert MTB/RIF testing for diagnosis of TB cases [15]. Subsequently, due to financial support provided by the Global Fund, GeneXpert MTB/RIF testing began in 2013 with the installation of hundreds of GeneXpert modules in TB laboratories across China [16]. However, the current low representation of GeneXpert-equipped prefecture-level laboratories highlights the need for better coordination of resource allocation between the public CDC sector and TB-designated hospitals. Third, another obvious challenge is the lack of external quality control efforts to validate results of various laboratory tests performed in TB-designated hospitals. Indeed, according to our results, approximately one quarter of TB laboratories do not incorporate external quality control measures for validating smear microscopy, phenotypic DST, and molecular test results. Therefore, measures should be taken to ensure that quality control systems are in place for delivery of high-quality laboratory services by TB-designated hospitals.

Management of TB patients is important for improving clinical outcomes during long-term treatment, since improper treatment management can lead to treatment failure and to emergence of drug resistance [17]. Interestingly, this study shows that TB-designated hospitals and local CDCs have made comparable contributions toward overall regular patient follow-up, in spite of national guidelines recommendations intended to steer directly observed therapy (DOT) delivery away from TB hospitals, where DOT is impractical due to human resource shortages. Recently, several novel electronic reminder and monitoring technologies have been under development or are currently under evaluation as alternatives to conventional DOT, such as mobile phone text messaging, electronic medication packaging devices, etc. [17, 18]. In fact, a series of reports have suggested that these innovative approaches hold promise for improving TB patient treatment adherence, especially in settings where universal administration of DOT is not feasible [17, 19]. In addition, we have found that TB cases seeking health care in four hospitals were lost to follow-up, putting those patients at high risk for poor treatment outcomes and contributing to amplification of drug resistance and community transmission [20]. Thus, an appropriate mechanism for TB case referral should be established to ensure proper case management to maximize desired treatment outcomes for high-risk patients. Taken together, our results have important implications for the present discussion about health-system reforms and their effects on TB control. Given the increasing involvement of TB-designated hospitals in public health affairs, additional government financing to provide resources to meet increased workloads would improve clinical management of TB cases to ultimately provide a high quality of care.

This study had several obvious limitations. First, only basic information was obtained from county-level hospitals, which made it difficult to study impacts of human resources, hospital revenues and expenditures, and laboratory workload levels of TB-designated hospitals. Second, the data were restricted to information collected by the questionnaires as well as to difficulties in locating staff for interviews. Despite potential biases, we feel that these biases do not diminish our key findings and conclusions. Finally, while HIV infection is known to exacerbate TB epidemics [21], we did not collect information regarding HIV status, since written informed consent is required prior to assessment of HIV status for each patient. Third, TB patient treatment outcomes are important for assessing the contribution of TB-designated hospitals to the overall impact of TB control programs in China. Unfortunately, such data were not collected in this study. Further studies are urgently needed to evaluate diagnostic and treatment practices in TB-designated hospitals and how they impact treatment outcomes.

In conclusion, this study describes dynamic changes in characteristics of TB-designated hospitals in China by comparing survey results obtained in 2015 with those obtained in 2009. The results demonstrate that China's health reform efforts have improved TB patient access to quality health care, although more attention is needed to address the unbalanced distribution of medical facilities across different regions of the country. In addition, laboratory capabilities and use of quality control systems should be enhanced to ensure delivery of high-quality laboratory services by TB-designated hospitals. Given the increasing involvement of TB-designated hospitals in public health affairs, the greater apportioning of government funding to areas of highest TB incidence and prevalence is required to improve overall clinical practices, quality of care, and TB case management in China.

## Figures and Tables

**Table 1:** Distribution of TB-designated hospitals stratified to geographic regions of China.

Region	County level			Prefecture level and above		
	No. of TB hospitals	No. of counties	Percentage of counties with TB hospitals (%)	No. of TB hospitals	No. of prefectures	Percentage of prefectures with TB hospitals (%)
Eastern	464	890	52.1	128	182	70.3
Central	435	888	49.0	109	113	96.5
Western	436	1091	40.0	113	197	57.4
Total	1335	2869	46.5	350	492	71.1

**Table 2:** Distribution of TB beds in the different regions of China.

Region	No. of population in 2010^a^	No. of TB beds	TB beds per 100,000 population	Prevalence of TB in 2010 per 100,000 population^b^	TB beds per 1,000 estimated TB cases
Eastern	549,937,502	12,664	2.30	291	7.91
Central	422,515,600	11,762	2.78	463	6.01
Western	360,357,767	7,677	2.13	695	3.07
Total	1,332,810,869	32,103	2.41	459	5.25

^a^Data source: China's Sixth National Population Census in 2010.

^b^Data source: China's Fifth National Tuberculosis Prevalence Survey in 2010.

**Table 3:** Comparison of laboratories in the prefectural tuberculosis-designated hospitals between 2009 and 2015.

Characteristics	2009	2015	Change
	(*n*=203)	(*n*=350)	(%)
*Smear microscopy*			
No. of tuberculosis-designated hospitals with testing capacity (%)	198 (98%)	334 (95%)	69%
Overall test volume	1,710,841	3,622,104	112%
Average no. of tests per tuberculosis-designated hospitals	8,641	10,845	26%
*Culture*			
No. of tuberculosis-designated hospitals with testing capacity (%)	128 (63%)	241 (69%)	88%
Overall test volume	275,476	898,176	226%
Average no. of tests per tuberculosis-designated hospitals	2,152	3,727	73%
*Phenotypic drug susceptibility testing*			
No. of tuberculosis-designated hospitals with testing capacity (%)	125 (62%)	203 (58%)	62%
Overall test volume	53,518	626,832	1071%
Average no. of tests per tuberculosis-designated hospitals	428	3,088	621%
*Molecular testing (except GeneXpert)*			
No. of tuberculosis-designated hospitals with testing capacity (%)	-	60 (17%)	-
Overall test volume	-	172,632	-
Average no. of tests per tuberculosis-designated hospitals	-	2,031	-
*GeneXpert*			
No. of tuberculosis-designated hospitals with testing capacity (%)	-	85 (24%)	-
Overall test volume	-	33,264	-
Average no. of tests per tuberculosis-designated hospitals	-	554	-

**Table 4:** Quality control of smear microscopy in the tuberculosis-designated hospitals.

Laboratory Method	Region	No. of TB-designated hospitals with testing capacity	No. of TB-designated hospitals with quality control	Percentage of TB-designated hospitals with quality control
Smear microscopy (County)	Eastern	475	408	87%
	Central	445	333	76%
	Western	336	238	71%
	Total	1256	979	79%

Smear microscopy (Prefecture)	Eastern	132	106	80%
	Central	118	78	66%
	Western	84	50	60%
	Total	334	234	70%

Phenotypic DST (Prefecture)	Eastern	105	88	84%
	Central	76	53	70%
	Western	22	14	64%
	Total	203	155	76%

Molecular method (Prefecture)	Eastern	45	33	73%
	Central	35	23	66%
	Western	18	10	56%
	Total	98	66	67%

**Table 5:** Management of tuberculosis patients in 350 prefectural TB-designated hospitals.

Type	No. of hospitals (%)			Total
	Eastern	Central	Western	
TB-designated hospital	28(21.88)	22(20.18)	26(23.01)	76(21.71)
Local CDC	35(27.34)	22(20.18)	24(21.24)	81(23.14)
Mixed pattern of hospital and CDC^a^	63(49.22)	63(57.80)	63(55.75)	189(54.00)
Loss to follow-up	2(1.56)	2(1.83)	0(0.00)	4(1.14)
Total	128(100.0)	109(100.0)	113(100.0)	350(100.0)

^a^Mixed pattern of hospital and CDC represents that the patients within the area of responsibility were managed by TB-designated hospital, whereas the other patients were transferred to local CDC.

## Data Availability

The authors declare that the data supporting the conclusions of this article are fully described within the article. The data that support the findings of this study are available from the corresponding authors upon request.
